# Supplementary material for: Phosphate starvation response precedes abscisic acid response under progressive mild drought in plants
Source: Nat Commun. 2023 Aug 19;14:5047. doi: 10.1038/s41467-023-40773-1 (PMC10439899; doi:10.1038/s41467-023-40773-1)
Supplement: Supplementary file 1 — Supplementary Information [file 41467_2023_40773_MOESM1_ESM.pdf]

Supplementary Information

Phosphate starvation response precedes abscisic acid response under progressive mild drought in plants

Yukari Nagatoshi, Kenta Ikazaki, Yasufumi Kobayashi, Nobuyuki Mizuno, Ryohei Sugita, Yumiko Takebayashi, Mikiko Kojima, Hitoshi Sakakibara, Natsuko I. Kobayashi, Keitaro Tanoi, Kenichiro Fujii, Junya Baba, Eri Ogiso-Tanaka, Masao Ishimoto, Yasuo Yasui, Tetsuji Oya, Yasunari Fujita*

*Corresponding author. E-mail: yasuf@affrc.go.jp

Supplementary Fig. 1

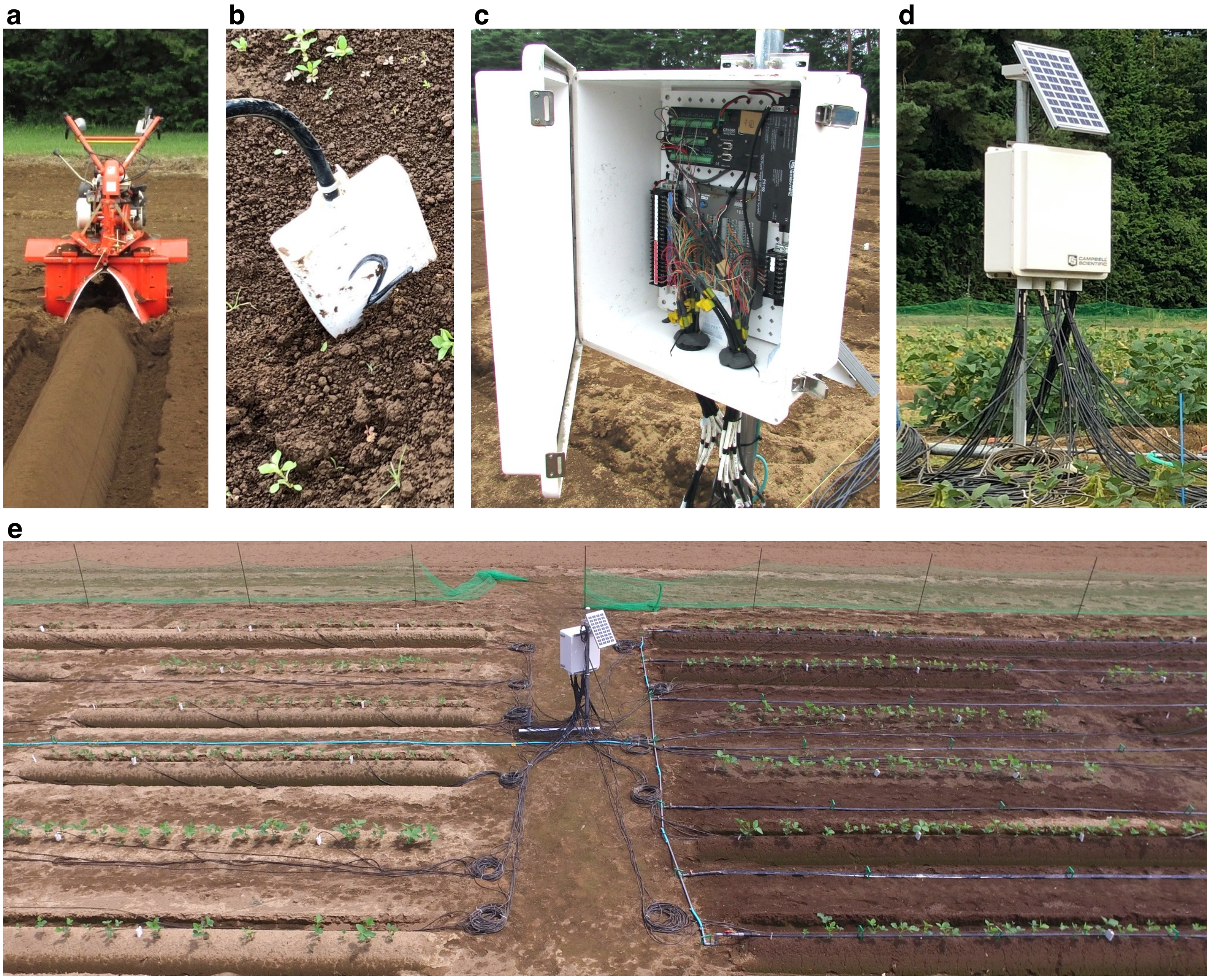

Supplementary Fig. 1. Experimental field plot, equipped with soil moisture sensors and an irrigation system, for analysis of ridge-induced mild drought.

a Ridge made using a tiller coupled to a rotary used in this study. **b** Time domain reflectometry (TDR) probes inserted into the soil to measure volumetric water content (VWC) over time during the cultivation period. **c, d** Soil VWC data collected from TDR probes inserted into the soil were sent to a Campbell CR1000 data logger (**c**) through coaxial cables (black cables) in a VWC data collection station (white box) powered by a solar panel (**d**). **e** The VWC data collection station was installed on a tower in the center of the experimental field equipped with an irrigation system. The area on the right was irrigated, whereas that on the left was not.

Supplementary Fig. 2

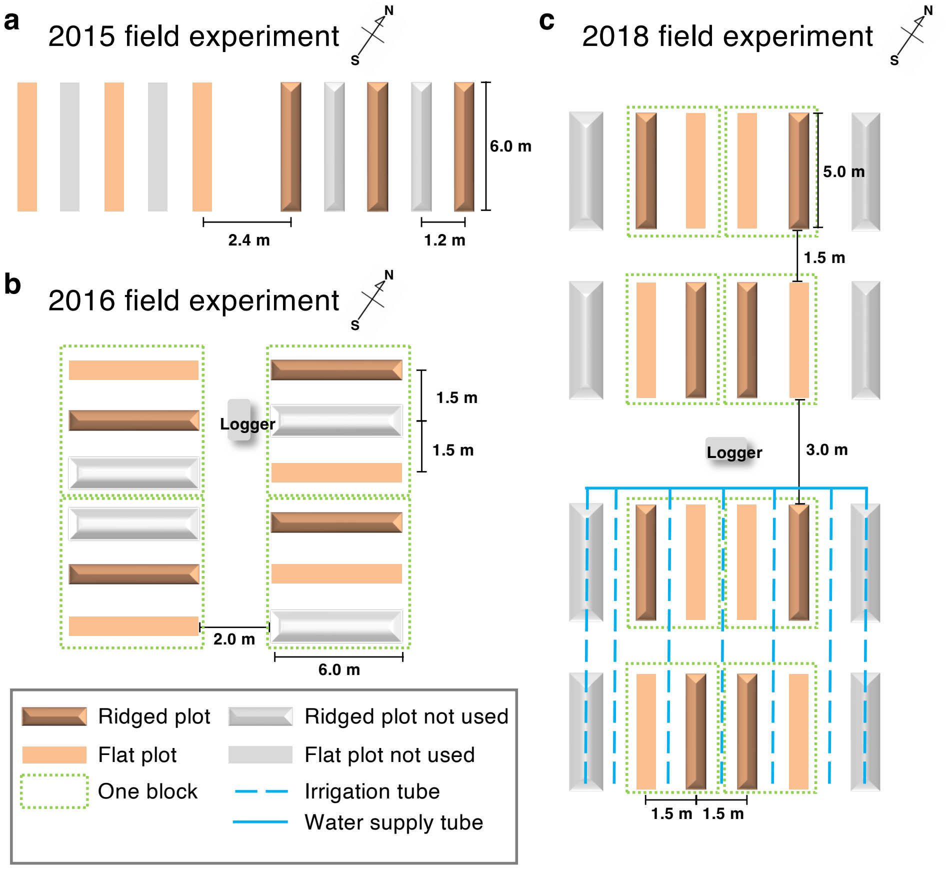

Supplementary Fig. 2. Schematic layouts of the experimental plots in 2015, 2016, and 2018. **a** In 2015, the experimental layout consisted of two areas, one with flat plots (left) and the other with ridged plots (right). In each area, three plots were used for the analysis. The other two plots in each area were not analyzed in this study. **b** In 2016, the experiment was laid out in a randomized complete block design, including flat and ridged plots, with four blocks for four replicates. Each block included a wider ridged plot that was not analyzed in this study. To monitor soil VWCs over time, TDR probes and a VWC data collection station with a built-in data logger were installed from 2016 onwards (Supplementary Fig. 1). **c** In 2018, the layout consisted of two separate areas, one irrigated and the other exclusively rainfed. Each area consisted of randomized blocks, each including flat and ridged plots, with four replicates. Wider ridges were constructed along the length of each block, on either side, and were not analyzed in this study.

Supplementary Fig. 3

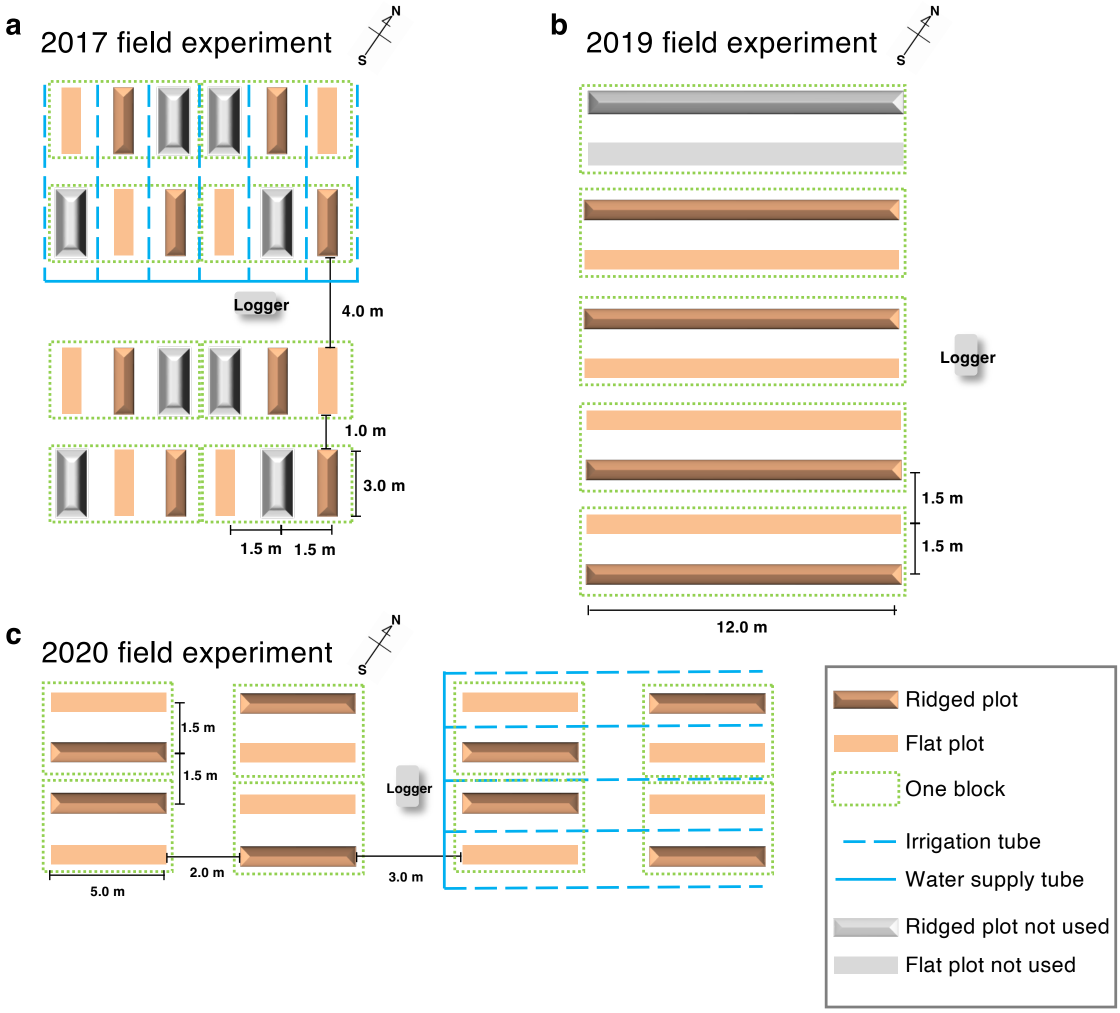

Supplementary Fig. 3. Schematic layouts of the experimental plots in 2017, 2019, and 2020.

a In 2017, the experiment layout was divided into two areas, one irrigated and the other exclusively rainfed. Each area included randomized blocks with flat and ridged plots, and with four replicates. Each block included a ridged plot with a wider base, which was not analyzed in this study. **b** In 2019, the experiment was laid out in a randomized block design, including flat and ridged plots, with four blocks for four replicates. The upper side of one block contained flats and ridged plots that were not analyzed in this study. **c** In 2020, the experimental layout was divided into two areas, one irrigated and the other exclusively rainfed. Each area included randomized blocks, including flat and ridged plots, with four replicates.

Supplementary Fig. 4

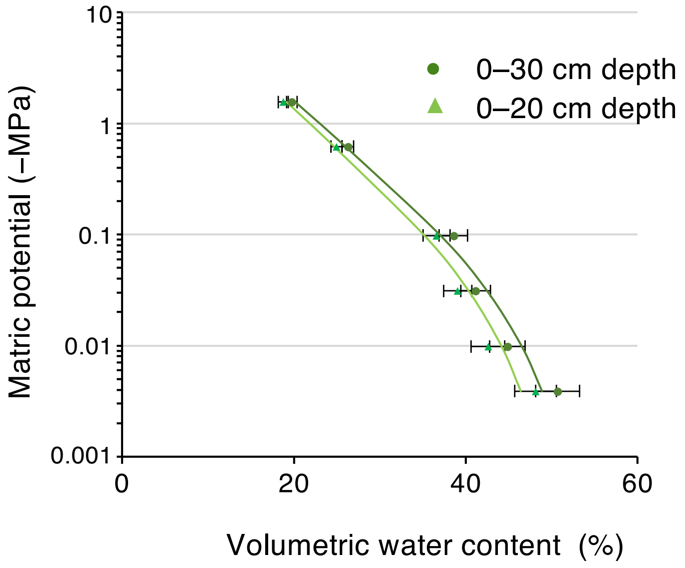

Supplementary Fig. 4. Relationship between VWC and matric potential of the soil in the experimental field.

Soil samples collected from the experimental field were used to measure the soil water retention. Dark green dots and light green triangles indicate the matric potential at a depth of 0–30 cm (BD value = 0.773) and 0–20 cm (BD value = 0.734), respectively (n = 6 independent replicates). For soil VWCs, VWCs at depths of 0–20 cm, starting from the top of the ridge or the flat, were measured in the 2015 field trials, and VWCs at depths of 0–30 cm, starting from the top of the ridge or the flat, were measured in the 2016 and subsequent field trials. Error bars denote SD.

Supplementary Fig. 5

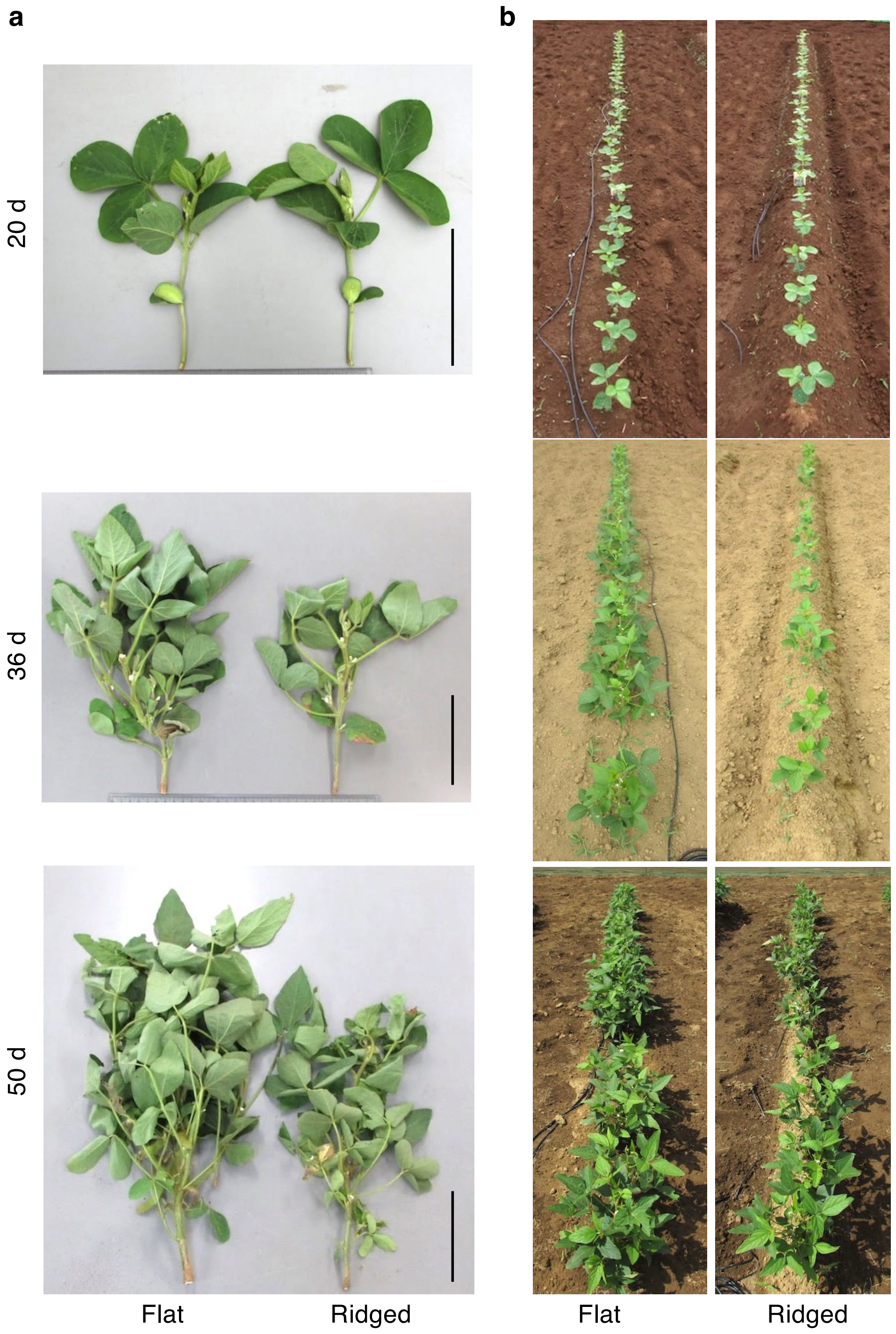

Supplementary Fig. 5. Growth of soybean plants on the flat and ridged plots at various growth stages in the 2016 field experiment.

a Representative 20-, 36-, and 50-day-old soybean plants grown on flat and ridged plots. Bars, 10 cm. **b** Representative 20-, 36-, and 50-day-old soybean plants in the field, on flat and ridged plots. Differences in soil color reflect the soil moisture at the time of sampling.

Supplementary Fig. 6

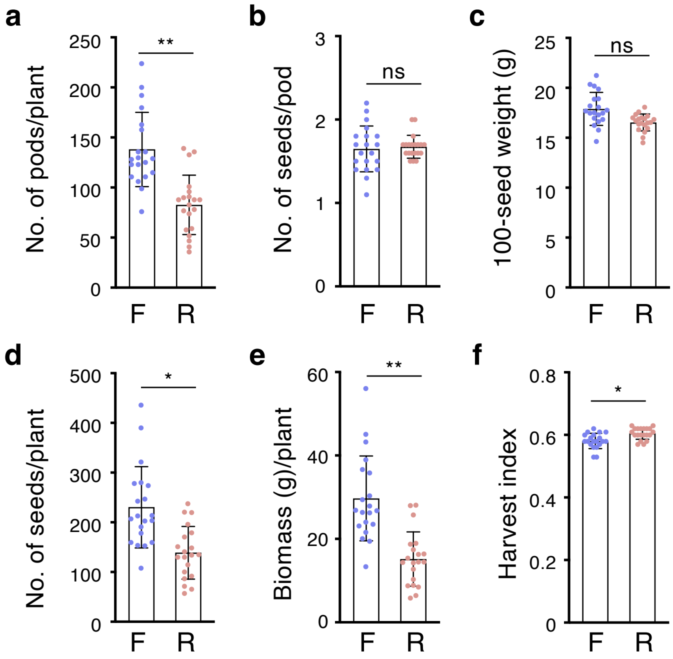

Supplementary Fig. 6. Yield components of soybean grown on flat and ridged plots in the 2016 field experiment.

a-f Measured values of yield components of soybean grown on flat and ridged plots in the 2016 field experiment. F and R denote flat and ridged plots, respectively. Number of pods per plant (**a**), number of seeds per pod (**b**), 100-seed weight (**c**), number of seeds per plant (**d**), dried aboveground biomass consisting of stem and pods (**e**), and calculated values of harvest index (**f**) of plants grown on the flat and ridged plots (n = 20 biologically independent replicates). $^{*}P < 0.05$, $^{**}P < 0.01$, $^{***}P < 0.001$, two-tailed paired samples t-test; ns, no significant difference. Error bars denote SD.

Supplementary Fig. 7

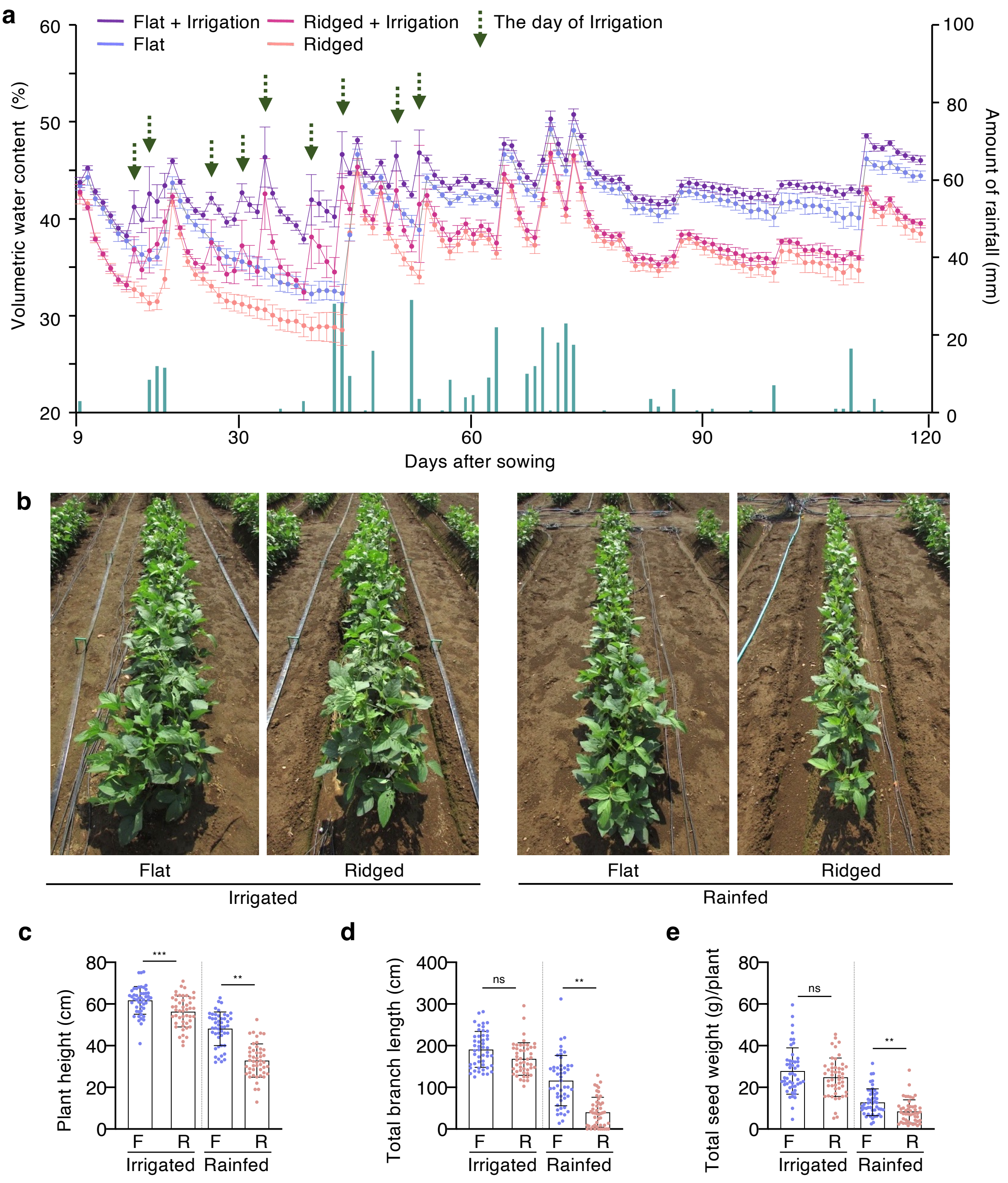

Supplementary Fig. 7. Irrigation compensated for the reduced growth of plants on ridges in the 2018 field experiment.

a Daily rainfall (green bars) and time course of the soil VWCs (n = 4 independent replicates) in the flat (blue and purple lines and points) and ridged (pink and red lines and points) plots with (purple and red lines and points) and without (blue and pink lines and points) irrigation over the growing period in 2018. Dashed green arrows indicate the day of irrigation. The relationships between soil VWC and water potential in the graphs were 50.7% (−0.0039 MPa, pF 1.6), 44.9% (−0.0098 MPa, pF 2.0), 41.2% (−0.031 MPa, pF 2.5), 38.6% (−0.098 MPa, pF 3.0), and 26.3% (−0.61 MPa, pF 3.8). **b** Forty-nine-day-old soybean plants on the flat and ridged plots with and without irrigation. **c-e** Plant height (**c**), total branch length (**d**), and total seed weight per plant (**e**) of the plants grown on the flat and ridged plots with (irrigated) and without (rainfed) irrigation (n = 48 biologically independent replicates). F and R denote flat and ridged plots, respectively. $^{**}P < 0.01$, $^{***}P < 0.001$, two-tailed paired samples t-test; ns, no significant difference. Error bars in (**a**), (**c**), (**d**), and (**e**) denote SD.

Supplementary Fig. 8

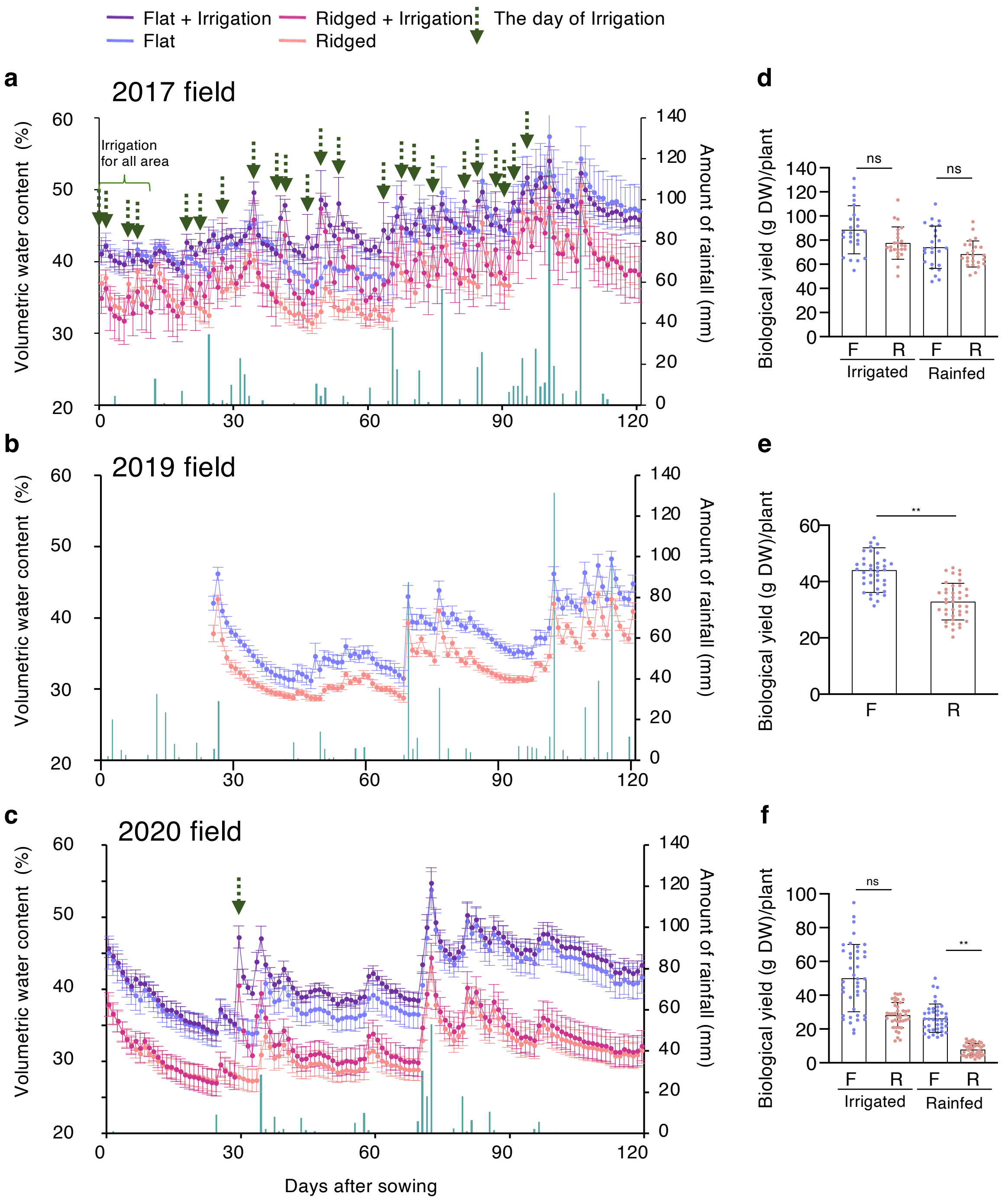

Supplementary Fig. 8. Ridges reduce soil water contents in the field.

a-c Daily rainfall (green bars) and time course of the soil VWCs (n = 4 independent replicates) in the flat (blue and purple lines and points) and ridged (pink and red lines and points) plots with (purple and red lines and points) and without (blue and pink lines and points) irrigation over the growing period in 2017 (**a**), 2019 (**b**), and 2020 (**c**). The relationships between soil VWC and water potential in the graphs were 50.7% (−0.0039 MPa, pF 1.6), 44.9% (−0.0098 MPa, pF 2.0), 41.2% (−0.031 MPa, pF 2.5), 38.6% (−0.098 MPa, pF 3.0), and 26.3% (−0.61 MPa, pF 3.8). Dashed green arrows indicate the day of irrigation. Error bar = SD. **d-f** The yield or biomass of the harvested soybean grown on flat (blue dots) and ridged (pink dots) plots with (irrigated) or without (rainfed) irrigation. F and R denote flat and ridged plots, respectively. n = 24 biologically independent replicates (**d**), n = 40 biologically independent replicates (**e, f**). Error bars denote SD. $^{**}P < 0.01$, $^{***}P < 0.001$, two-tailed paired samples t-test; ns, no significant difference.

Supplementary Fig. 9

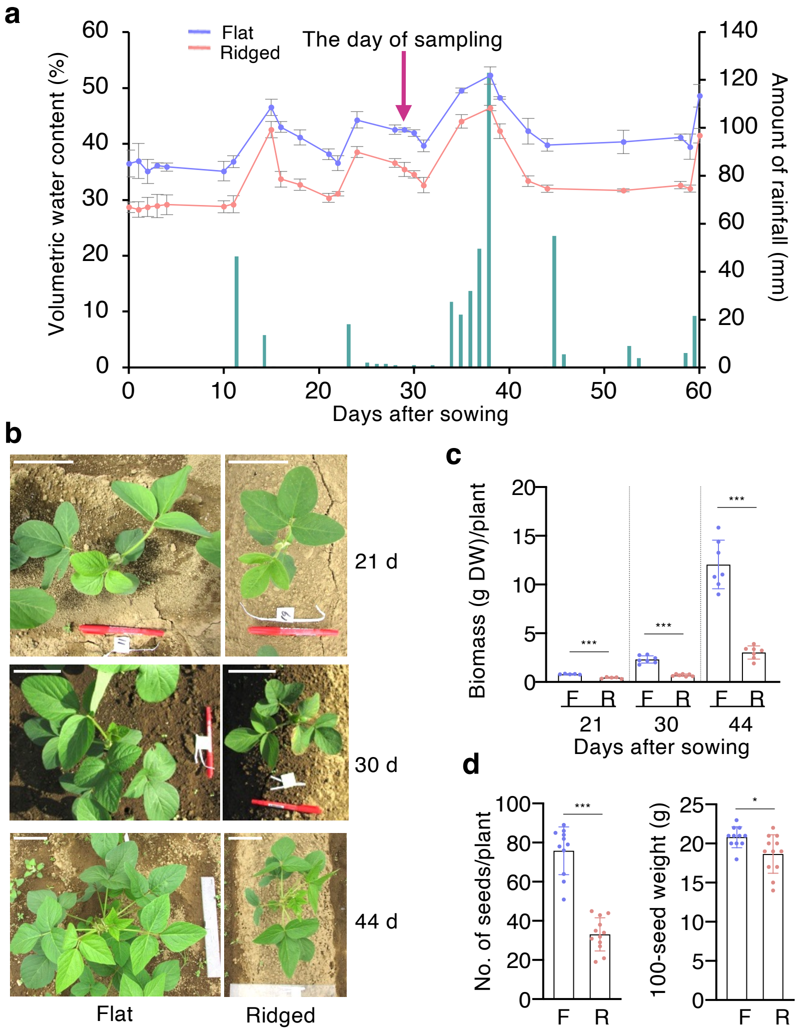

Supplementary Fig. 9. **Ridges induced mild drought and hampered soybean growth in the 2015 field experiment.**

a Daily rainfall (green bars) and time course of the soil VWCs (n = 3 independent replicates) in the flat (blue lines and dots) and ridged (pink lines and dots) plots over the growing period in the 2015 field. Magenta arrow indicates the day of sampling for RNA-seq analysis. The relationships between soil VWC and water potential in the graphs were 48.1% (−0.0039 MPa, pF 1.6), 42.6% (−0.0098 MPa, pF 2.0), 39.1% (−0.031 MPa, pF 2.5), 36.6% (−0.098 MPa, pF 3.0), and 25.0% (−0.61 MPa, pF 3.8). **b** Representative 21-, 30-, and 44-day-old soybean plants on the flat and ridged plots. Bars, 10 cm. **c** Aboveground biomass (dry weight) per plant of 21-, 30-, and 44-day-old plants grown on the flat and ridged plots (n = 5 biologically independent replicates for the samples of 21-day-old plants; 7 biologically independent replicates for the samples of 30- and 44-day-old plants). F and R denote flat and ridged plots, respectively. **d** Number of seeds per plant (left) and 100-seed weight (right) of the harvested plants grown on the flat and ridged plots (n = 11 biologically independent replicates for the flat samples; n = 12 biologically independent replicates for the ridged samples). $^{*}P < 0.05$, $^{***}P < 0.001$, two-tailed Student's t-test. Error bars in (**a**), (**c**), and (**d**) denote SD.

Supplementary Fig. 10

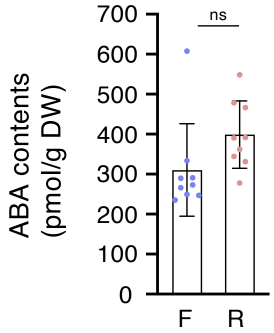

Supplementary Fig. 10. **ABA content did not clearly differ between the leaves of soybean plants grown on flats and ridges.**

ABA contents of second trifoliate leaves of soybean plants grown on flats (F) and ridges (R) (*n* = 9 biologically independent replicates); the same samples were used for the RNA-seq analysis presented in Fig. 3. ns indicates not significant, two-tailed Student's *t*-test. Error bars denote SD.

Supplementary Fig. 11

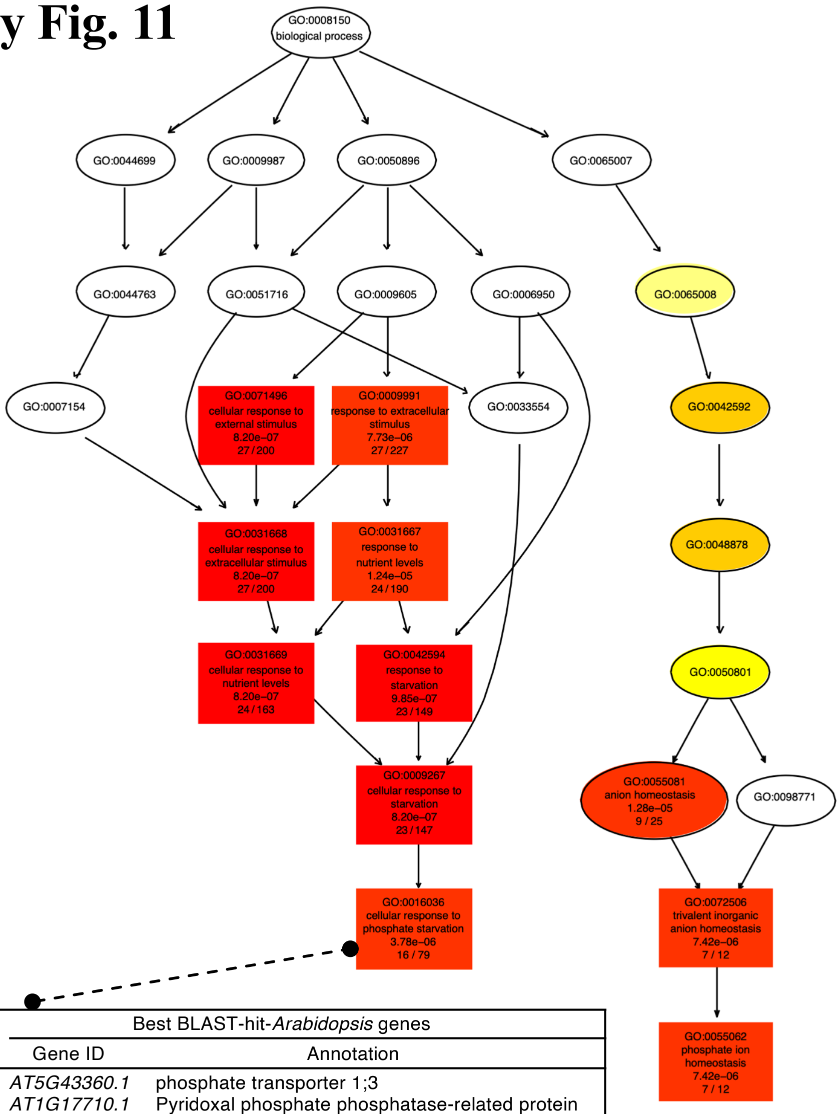

	Best BLAST-hit-*Arabidopsis* genes	
Gene ID	Gene ID	Annotation
Glyma.10G006700	*AT5G43360.1*	phosphate transporter 1;3
Glyma.08G195000	*AT1G17710.1*	Pyridoxal phosphate phosphatase-related protein
Glyma.07G011900	*AT1G17710.1*	Pyridoxal phosphate phosphatase-related protein
Glyma.03G162800	*AT3G54700.1*	phosphate transporter 1;7
Glyma.06G028200	*AT2G27190.1*	purple acid phosphatase 12
Glyma.13G270400	*AT3G14940.1*	phosphoenolpyruvate carboxylase 3
Glyma.07G222700	*AT1G76430.1*	phosphate transporter 1;9
Glyma.13G351600	*AT1G17710.1*	Pyridoxal phosphate phosphatase-related protein
Glyma.10G166600	*AT1G08650.1*	phosphoenolpyruvate carboxylase kinase 1
Glyma.13G161900	*AT5G34850.1*	purple acid phosphatase 26
Glyma.03G251400	*AT3G04530.1*	phosphoenolpyruvate carboxylase kinase 2
Glyma.20G222600	*AT1G08650.1*	phosphoenolpyruvate carboxylase kinase 1
Glyma.19G200000	*AT3G11670.1*	UDP-Glycosyltransferase superfamily protein
Glyma.12G007500	*AT2G16430.2*	purple acid phosphatase 10
Glyma.10G186500	*AT3G54700.1*	phosphate transporter 1;7
Glyma.10G186400	*AT2G38940.1*	phosphate transporter 1;4

	Best BLAST-hit-*Arabidopsis* genes	
Gene ID	Gene ID	Annotation
Glyma.05G138400	*AT2G01890.1*	purple acid phosphatase 8
Glyma.06G028200	*AT2G27190.1*	purple acid phosphatase 12
Glyma.16G052000	*AT5G41080.1*	PLC-like phosphodiesterases superfamily protein
Glyma.13G161900	*AT5G34850.1*	purple acid phosphatase 26
Glyma.08G093500	*AT1G14700.1*	purple acid phosphatase 3
Glyma.09G223700	*AT3G47420.1*	phosphate starvation-induced gene 3
Glyma.12G007500	*AT2G16430.2*	purple acid phosphatase 10

Supplementary Fig. 11. Mild drought induces the expression of many PSR genes in soybean plants in the field.

Gene Ontology (GO) term enrichment analysis of genes up-regulated in soybean under ridge-induced mild drought conditions. GO analysis (FDR < 0.05, $p < 0.05$) revealed a total of 53 GO terms, mainly involving stress-responsive genes, that were significantly enriched in up-regulated DEGs. Notably, among the up-regulated DEGs, 16 and 7 were related to “cellular response to phosphate starvation” and “phosphate ion homeostasis”, respectively. The enriched GO terms are visualized in a directed acyclic graph (DAG) with colors representing the p value (red shows a lower p value than yellow).

Supplementary Fig. 12

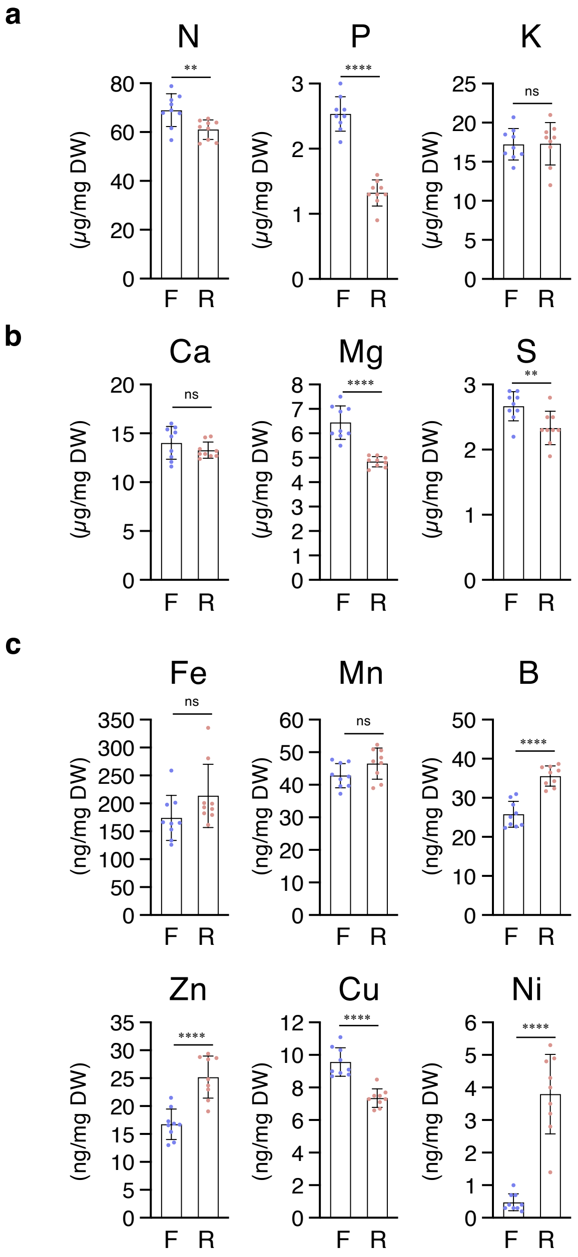

Supplementary Fig. 12. Elemental analysis shows a marked decrease in P in plant leaves under mild drought.

The contents of primary macronutrients (**a**), secondary macronutrients (**b**), and micronutrients (**c**) in second trifoliate leaves of soybean plants grown on flats and ridges; the same samples were used for the RNA-seq analysis presented in Fig. 3. F and R denote flat and ridged plots, respectively (n = 9 biologically independent replicates). $^{**}P < 0.01$, $^{***}P < 0.001$, and $^{****}P < 0.0001$; ns indicates not significant, two-tailed Student's t-test. Error bars denote SD.

Supplementary Fig. 13

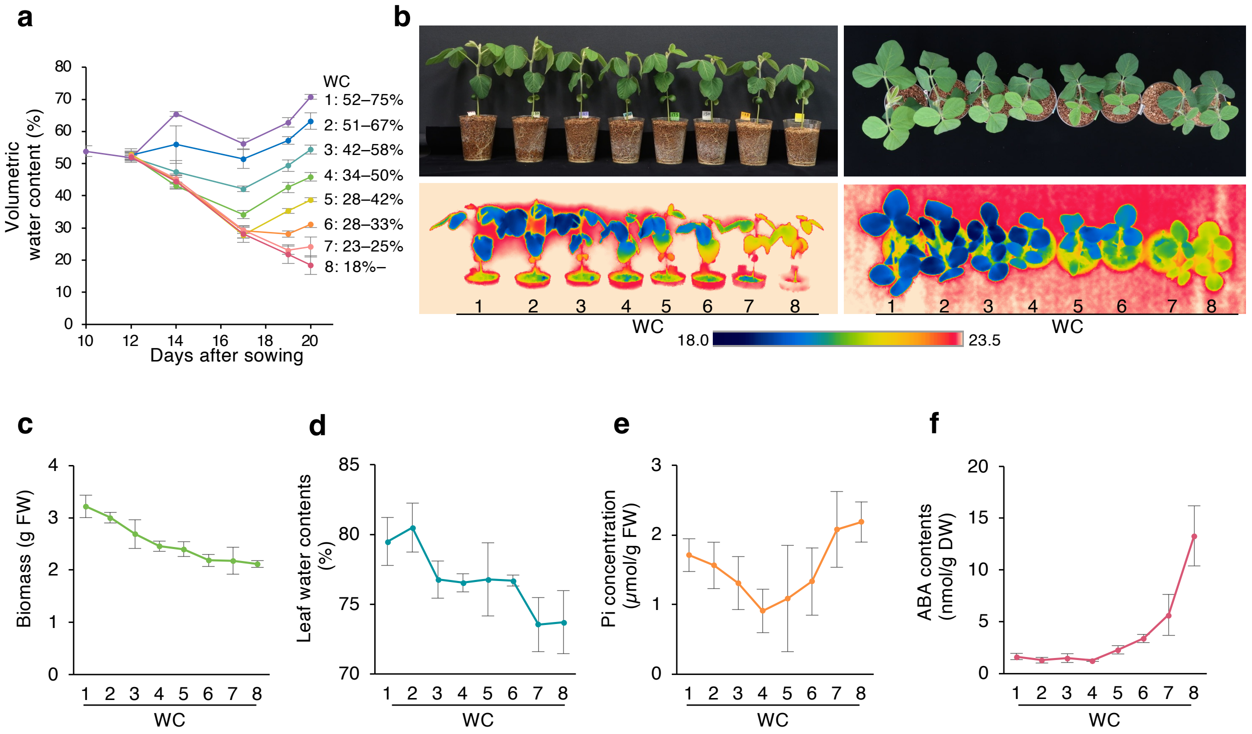

Supplementary Fig. 13. Mild drought reduces Pi and severe drought increases ABA in pot-grown soybean plants.

a Time-course analysis of VWCs in potted soybean plants with eight different moisture contents (n = 8 independent replicates). The range of variation in VWCs during the period from day 17 after sowing to day 20 (when sampling was performed) is shown on the right. Drought stress treatments were imposed from 12 days after sowing. The relationships between soil VWC and water potential in the graphs were 36.0% (−0.0039 MPa, pF 1.6), 32.5% (−0.0098 MPa, pF 2.0), 25.8% (−0.031 MPa, pF 2.5), 16.9% (−0.098 MPa, pF 3.0), and 3.9% (−0.61 MPa, pF 3.8). **b** Thermogram and corresponding digital image of 20-day-old soybean plants in pots viewed from the side and top. **c-f** Aboveground biomass (c), leaf water content (d), Pi concentration (e), and ABA content (f) of the second trifoliate leaves (n = 4 biologically independent replicates). Error bars denote SD.

Supplementary Fig. 14

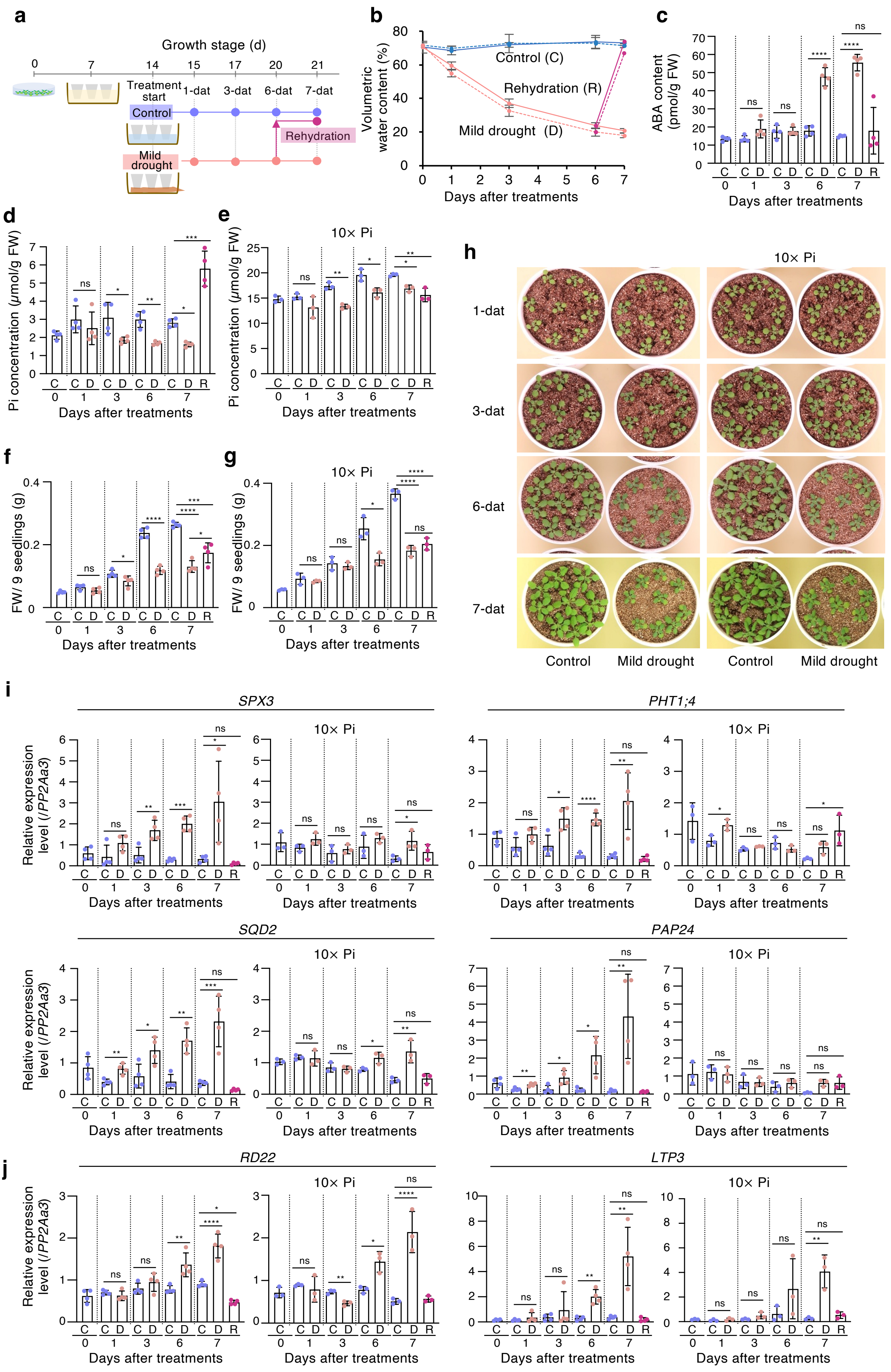

Supplementary Fig. 14. PSR induced by Pi reduction initially occurs before ABA response under progressive mild drought in pot-grown *Arabidopsis* plants.

a Schematic overview of the experimental design for control, mild drought, and rehydration treatment in *Arabidopsis* plants. Pots containing 14-day-old seedlings soaked in water with control liquid nutrients or liquid nutrients with excess Pi (10 × Pi: 10 times the amount of Pi) after transplanting 7-day-old plants from agar plates were placed on paper towels for mild drought treatment, soaked in water for the control, or placed on paper towels for 6 days and then soaked in water for 1 day for the rehydration treatment. The plants were sampled at 1, 3, 6, and 7 days after treatment (dat). **b** VWCs at treatment initiation and sampling in pots. The solid line indicates control nutrients (n = 4 independent replicates), and the dotted line indicates liquid nutrients with 10 × Pi (n = 3 independent replicates). Relationships between the average of soil VWC and water potential were control treatment (71.6%, >−0.0031 MPa), mild drought 1-dat (57.2%, > −0.0031 MPa), mild drought 3-dat (34.9%, −0.0079 MPa), mild drought 6-dat (21.9%, −0.19 MPa), and mild drought 7-dat (19.4%,−0.25 MPa). **c** ABA contents of the seedlings grown in pots soaked in water with control liquid nutrients (n = 4 independent pot replicates, three seedlings per pot). **d, e** Pi concentrations of the seedlings grown in pots soaked in water with control liquid nutrients (**d**, n = 4 independent pot replicates, three seedlings per pot) and with liquid nutrients with 10 × Pi (**e**, n = 3 independent pot replicates, three seedlings per pot). The values on the vertical axis differ for control liquid nutrients and liquid nutrients with 10 × Pi. **f, g** Total aboveground biomass (fresh weight, FW) per pot; nine plants were grown per pot in control liquid nutrients (**f**, n = 4 independent pot replicates) and liquid nutrients with 10 × Pi (**g**, n = 3 independent pot replicates). **h** Photographs of *Arabidopsis* seedlings at 1, 3, 6, and 7 days after control and mild drought treatment in pots pre-soaked in control liquid nutrients and liquid nutrients with 10 × Pi. **i, j** Relative expression of PSR (**i**)- and ABA-responsive (**j**) genes in control liquid nutrients (n = 4 independent pot replicates) and liquid nutrients with 10 × Pi (n = 3 independent pot replicates), as determined by RT-qPCR. Expression was normalized to that of *PP2Aa3*. C, D, and R denote control, mild drought, and rehydration treatment, respectively. $^{*}P < 0.05$, $^{**}P < 0.01$, $^{***}P < 0.001$ and $^{****}P < 0.0001$; ns indicates not significant, two-tailed Student's t-test or one-way ANOVA with Tukey's test (for 7-dat). Error bars denote SD.

Supplementary Fig. 15

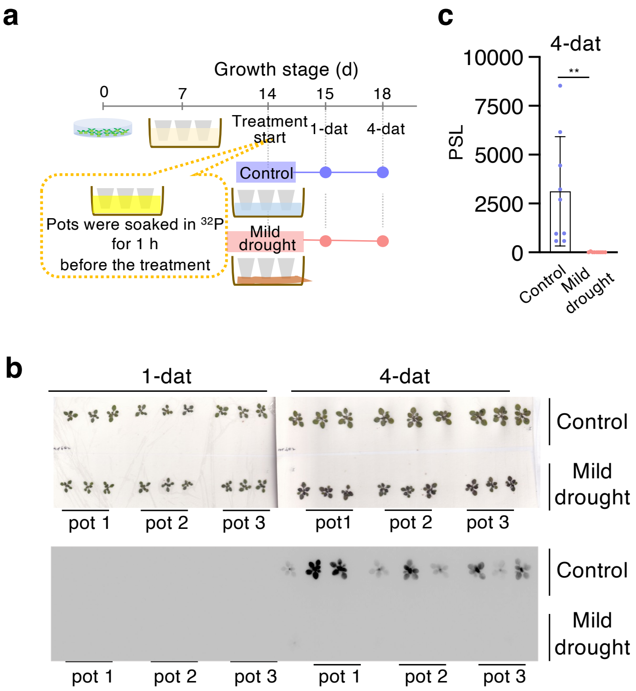

Supplementary Fig. 15. Mild drought inhibits Pi uptake into the aboveground parts of plants.

Evaluation of Pi uptake using *Arabidopsis* plants radiolabeled with ^{32}Pi. **a** Schematic overview of the experimental design for control and mild drought in *Arabidopsis* plants. Pots containing 14-day-old seedlings soaked in water with liquid nutrients after transplanting 7-day-old plants from agar plates were soaked in water containing ^{32}Pi for 1 h. The pots were placed on paper towels for the mild drought treatment or soaked in water for the control. The plants were sampled at 1 and 4 days after treatment (dat). **b** Autoradiography of the aboveground parts of the *Arabidopsis* plants radiolabeled with ^{32}Pi. **c** ^{32}P content of each plant (aboveground parts) calculated using a photostimulated luminescence (PSL) value and calibration curve based on the standard spots (n = 9 biologically independent replicates). $^{**}P < 0.01$, two-tailed Student's t-test. Error bars denote SD.

Supplementary Fig. 16

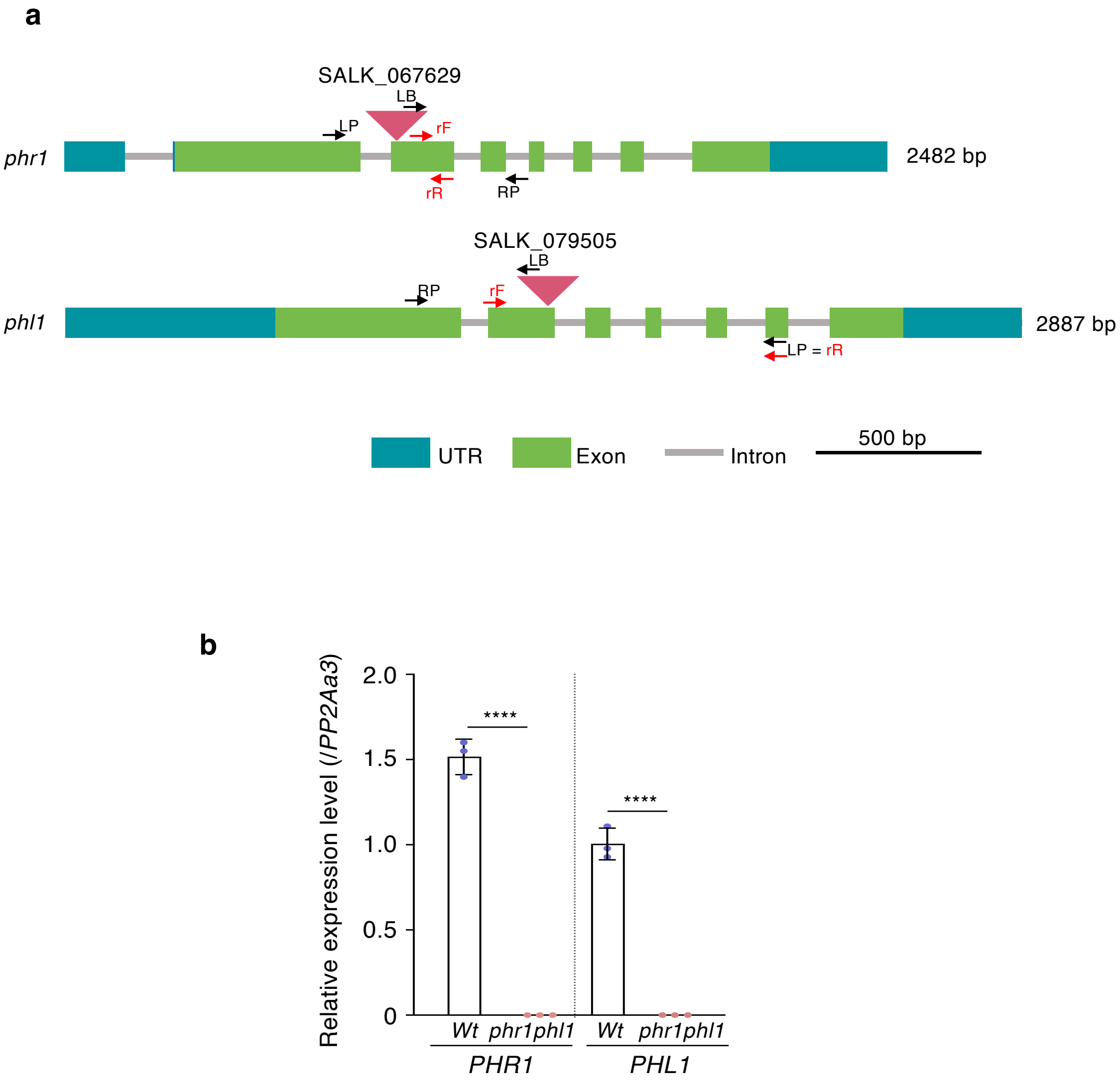

Supplementary Fig. 16. ***Arabidopsis phr1 phl1*** **double knockout mutant deficient in PSR.**
a Diagrams of *PHR1* and *PHL1* genes indicating the positions of T-DNA insertions. LB, LP and RP indicate the primer positions used to check the T-DNA insertions. rF and rR indicate the primer positions used to analyze the gene expression by RT-qPCR. **b** Relative expression levels of *PHR1* and *PHL1* genes in the *phr1 phl1* double knockout mutant were determined by RT-qPCR (n = 3 independent pot replicates, three seedlings per pot) with total RNA isolated from 18-day-old seedlings of the WT and *phr1 phl1* double mutant. Expression was normalized to that of *PP2Aa3*. $^{****}P < 0.0001$, two-tailed Student's t-test. Error bars denote SD.
