# Supplementary material for: Phosphate starvation response precedes abscisic acid response under progressive mild drought in plants
Source: Nat Commun. 2023 Aug 19;14:5047. doi: 10.1038/s41467-023-40773-1 (PMC10439899; doi:10.1038/s41467-023-40773-1)
Supplement: Supplementary file 3 — Description of Additional Supplementary Files [file 41467_2023_40773_MOESM3_ESM.pdf]

Description of Additional Supplementary Files:

Supplementary Data 1: List of differentially expressed genes (DEGs) between the soybean plants grown on flats and those grown on ridges.

Supplementary Data 2: List of up-regulated genes (|log2 (FC)| ≥ 1) amongst DEGs between the soybean plants grown on flats and those grown on ridges.

Supplementary Data 3: List of down-regulated genes (|log2 (FC)| ≥ 1) amongst DEGs between the soybean plants grown on flats and those grown on ridges.

Supplementary Data 4: List of GO analysis of up-regulated genes (|log2 (FC)| ≥ 1) amongst DEGs between the soybean plants grown on flats and those grown on ridges.

Supplementary Data 5: List of PSR genes amongst the up-regulated DEGs between the soybean plants grown on flats and those grown on ridges.

Supplementary Data 6: A literature-curated set of PSR marker genes in Arabidopsis and soybean.

Supplementary Data 7: Nutrient contents of the vermiculite used in this study.

Supplementary Data 8: List of differentially expressed genes between Arabidopsis thaliana under control and drought conditions after 1 day of treatment.

Supplementary Data 9: List of differentially expressed genes between Arabidopsis thaliana under control and drought conditions after 3 days of treatment.

Supplementary Data 10: List of differentially expressed genes between Arabidopsis thaliana under control and drought conditions after 6 days of treatment.

Supplementary Data 11: List of differentially expressed genes between Arabidopsis thaliana under control and drought conditions after 7 days of treatment.

Supplementary Data 12: List of ABA-responsive genes in Arabidopsis thaliana used in this study.

Supplementary Data 13: List of hierarchical clustering genes that were differentially expressed in response to mild drought stress in at least one sampling in the RNA-seq experiments.

Supplementary Data 14: Primer pairs used for quantitative real-time PCR in this study.
